# Sitting in Judgment: How Body Posture Influences Deception Detection and Gazing Behavior

**DOI:** 10.3390/bs11060085

**Published:** 2021-06-10

**Authors:** Mircea Zloteanu, Eva G. Krumhuber, Daniel C. Richardson

**Affiliations:** 1 Department of Psychology, Kingston University, London KT1 2EE, UK; 2 Department of Criminology and Sociology, Kingston University, London WC1H 0AP, UK; 3 Department of Experimental Psychology, University College London, London WC1H 0AP, UK; daniel.richardson@ucl.ac.uk

**Keywords:** accuracy, bias, body postures, deception detection, embodiment, empathy, experimental design, eye tracking, facial expressions, veracity judgment

## Abstract

Body postures can affect how we process and attend to information. Here, a novel effect of adopting an open or closed posture on the ability to detect deception was investigated. It was hypothesized that the posture adopted by judges would affect their social acuity, resulting in differences in the detection of nonverbal behavior (i.e., microexpression recognition) and the discrimination of deceptive and truthful statements. In Study 1, adopting an open posture produced higher accuracy for detecting naturalistic lies, but no difference was observed in the recognition of brief facial expressions as compared to adopting a closed posture; trait empathy was found to have an additive effect on posture, with more empathic judges having higher deception detection scores. In Study 2, with the use of an eye-tracker, posture effects on gazing behavior when judging both low-stakes and high-stakes lies were measured. Sitting in an open posture reduced judges’ average dwell times looking at senders, and in particular, the amount and length of time they focused on their hands. The findings suggest that simply shifting posture can impact judges’ attention to visual information and veracity judgments (M*_g_* = 0.40, 95% CI (0.03, 0.78)).

## 1. Introduction

People are poor at detecting if others are lying or telling the truth, usually being only slightly better than predicted by chance [1,2]. They are also overconfident in their judgments [3] and tend to assume most statements people make are honest, commonly referred to as the truth bias [4]. The majority of past research attempting to improve deception detection accuracy has focused either on differences between judges (e.g., experience or training; [5,6]) or between senders (e.g., demeanor or physical characteristics; [7,8]). Here, we raise the possibility that simply changing the judge’s body posture might affect their ability to perceive and interpret behavioral information from a sender.

Changes in posture appear to have systematic and causal effects on how we see the world and ourselves [9,10]. However, it is not yet clear whether posture has these effects by modulating attention, emotion, or some other cognitive processes. Here, we manipulated body posture while asking people to judge if others were lying or being honest, assessing how their emotional perception, visual attention, and veracity judgments were changed. In this context, our goal was to understand how postural changes might interact with complex social judgments.

### 1.1. Body Postures

Postures are representative of gross affect [11], reflecting dimensions such as friendliness and unfriendliness [12]. Body postures can affect how one perceives, reacts to, and interprets information [9]. Adopting specific postures can influence the likelihood that certain thoughts occur [10] and serve as a cue for how individuals view themselves and external stimuli [13,14,15,16].

Research on the psychological effects of posture is still in its infancy, and, at times, a controversial topic. For instance, the literature on “power posing”—adopting expansive postures to increase feelings of power [17]—has shown weaker and more limited effects in recent reexaminations [18,19].

In the past, research has linked the posture a person adopts with how they interact with the world. For instance, adopting an upright posture has been associated with improved mood, higher self-esteem, and increased arousal [20,21]. Posture can also affect information processing and attention. Riskind [22] reported quicker recall for pleasant memories when participants sat in an upright posture, but quicker recall for negative events (both in quantity and intensity) when in a slumped posture. Similar effects on cognition and memory have been found, where the posture adopted by an individual (upright or slumped) directly affected performance on valence word tasks [23] and mood-recovery interventions [24].

Our manipulation rests on open and closed postures. Open postures are expansive physical displays adopted by individuals when feeling relaxed and willing to engage in social interaction [25]; they can produce power-related feelings and cognitions [15]. Typically, an open posture has individuals sitting with their arms uncrossed and legs uncrossed, in a relaxed recline. A closed posture, in contrast, typically has individuals sitting with their arms and legs crossed, in a rigid position; it is adopted when feeling threatened or dismissive of another, signaling a lack of desire to interact. Adopting a closed posture increases one’s sensitivity to how others evaluate you [26] and can activate the behavioral inhibition system [27], resulting in reduced expressivity, gesturing, and contact with the environment [28]. These postures are argued to reflect the embodied concept of the “openness” dimension of social interaction, lying at opposite ends of the spectrum [12].

We propose that adopting a closed posture results in social information being ignored or processed to a lesser extent [29]. Conversely, an open posture reflects that the individual feels relaxed, and primes them to be open to interaction, preparing them to be more responsive and attentive to social cues [30].

### 1.2. Facial Expressions

When judging the veracity of others, people tend to rely on nonverbal behavior to help make their decisions [31,32]. Several prevalent cross-cultural beliefs exist about cues to detecting deceit, such as liars self-touch, avert their gaze, and fidget more than truth-tellers [32]. Regardless of their diagnostic value (which is usually quite poor; [33,34]), people’s beliefs regarding such nonverbal behaviors are strong [32].

As the current interest is on how postures can impact the decoding of behavioral cues, we focused on the recognition of brief facial expressions of emotions—microexpressions—proposed in the deception literature as a source of diagnostic information [35,36]. Microexpressions are involuntary full-faced expressions occurring at less than 0.5 of a second, said to reflect the genuine emotional state of the sender [35,37]. It is argued that liars and truth-tellers experience different emotions, which result in differences in the facial expressions each produces [35]. Thus, the assumption is that a perceptive judge can utilize these cues to accurately determine the veracity of a sender [35].

However, the literature on the emotion-based approach to lie detection is highly contentious [36,38]. While some studies indicate that, in specific scenarios, the ability to detect microexpressions is positively related to deception detection performance [39,40], more recent evaluations find no link between emotional cues and improvements in accuracy [41,42].

At present, the inclusion of a microexpression task was considered for two reasons. First, out of all nonverbal channels, facial expressions receive preferential attention and mental processing relative to other nonverbal channels [43,44]. If the posture manipulation has an effect on social acuity, a difference in facial expression recognition ability may be most evident. Second, while the evidence on the relationship between microexpression and deception detection is mixed (especially regarding human judges; [42]), people do hold strong beliefs that facial expressions are diagnostic of deceit [32,35]. If judges in a specific posture show a difference in veracity judgments (beneficial or otherwise), an overreliance on facial cues may explain the effect. Thus, if postures can affect people’s ability to perceive and/or interpret behavioral cues, facial expressions are an ideal candidate for measuring performance differences.

The posture effect was investigated over two experiments. In Study 1, the effect of adopting either an open or closed posture on veracity judgments was investigated. Consideration was also given to exploring the role of individual differences in trait empathy and the ability to recognize microexpressions. We predicted that adopting an open posture would result in more accurate veracity judgments, while increased nonverbal sensitivity to emotional stimuli (i.e., higher empathy and microexpression recognition) would bolster the effect. In Study 2, the framework for investigating the posture effect was expanded to consider the type of lie (either low-stakes or high-stakes), while also considering the effect of posture on gazing behavior towards senders, especially towards nonverbal channels of communication. Much of the emotion-based lie detection research argues that an increased number of nonverbal cues (i.e., in high-stakes scenarios) and attention to nonverbal channels (e.g., facial expressions) improves deception detection Thus, if posture influences social acuity, as presently proposed, judges adopting an open posture should attend more to nonverbal information and integrate it more optimally in their veracity judgments compared to the closed posture judges.

## 2. Study 1

The posture manipulation is predicted to impact social acuity, specifically the ability to perceive and interpret the behavioral information from senders. As such, we hypothesized the posture manipulation would also affect deception detection performance, as adopting such postures will impact the judges’ attention to and interpretation of the behavioral information displayed by liars and truth-tellers. As the posture effect is presumed to impact judgment through a change in attention to behavioral cues, this would be best reflected in the judges’ ability to detect and categorize the brief facial expressions of emotions (i.e., microexpressions). Thus, our second hypothesis was that posture would affect judges’ brief emotion recognition accuracy. As empathy can affect facial expression recognition [45,46] and deception detection [38,47], the differences in trait empathy were also considered as an explanatory variable.

## 3. Materials and Methods

### 3.1. Participants and Design

A mixed-design was used, with Posture (Open or Closed) serving as the between-subjects factor and message Veracity (Lies and Truths) as the within-subjects factor. The dependent variables were deception detection accuracy, judgment bias, and microexpression recognition. Individual differences in empathy were also measured to be used as a control variable in the subsequent analyses.

Eighty participants (30 males, 50 females; *M_Age_* = 25.30, *SD* = 8.49) were recruited through the university’s online subject pool, in return for GBP 1.00 or course credit. An a priori power analysis using G*Power 3.1 [48] for an interaction between Posture (2) and Veracity (2), assuming a medium-sized effect (Cohen’s *f* = 0.25), determined that a sample of this size would be sufficient for 95% power at the 0.05 alpha criterion.

### 3.2. Stimuli and Measures

Twelve videos (6 lies and 6 truths) from the Bloomsbury Deception Set were used (BDS; [49]). Senders in the videos are describing past vacations, where half of the stories are fabrications. The lies told are naturalistic, as the aim of the senders was to deceive the person recording the video, who was not told of the deception occurring; senders were not given any incentive to deceive beyond being asked to help with a travel documentary. The videos contained an equal number of male and female senders, and no sender was used twice. All videos are around 30 s in length. 

Facial recognition ability was assessed using the Micro Expression Training Tool (METT; [50]). The METT was developed to train the recognition of microexpressions of the seven basic emotions: happiness, anger, sadness, disgust, fear, surprise, and contempt. The test module consists of 14 color portrait photographs of facial expressions of emotions (Japanese and Caucasians), two for each emotion. Users view a neutral expression followed by an emotional expression for 1/25^th^ of a second, to which they respond with an emotion label from the list of seven displayed on the screen. The maximum test score is 100%. The METT has been used in past studies (e.g., [40,41,42]), and is based on the Brief Affect Recognition Test, which has good validity and reliability [51].

Empathy was measured using the Interpersonal Reactivity Index (IRI; [52]). The measure consists of 28 questions (e.g., “I really get involved with the feelings of the characters in a novel”), of which seven are specific to each of the four subscales: Perspective-taking, Fantasy, Empathic Concern, and Personal Distress. The IRI has high internal and external validity [53] and good test-retest reliability [52,54].

### 3.3. Procedure

**Figure 1:**
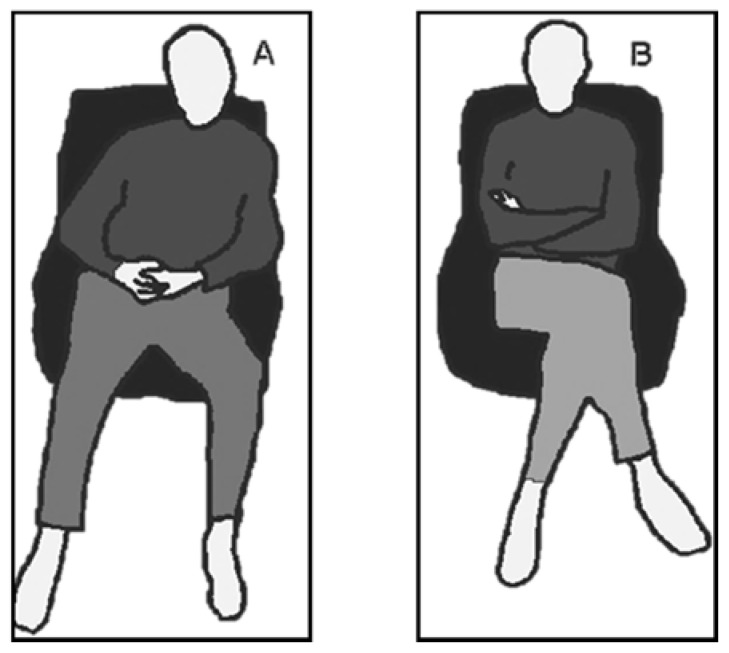
Visual depictions of the open (**A**) and closed (**B**) postures presented to participants.

Participants first completed the facial recognition task. Before the task began, they were given a demonstration of the software. They then completed the IRI. For each item, they responded using a scale with letters from A *(does not describe me well)* to E *(describes me very well)*. Afterward, participants were randomly allocated to either the Open or Closed posture condition and undertook the deception detection task. The experiment was presented as an ergonomics study interested in posture and task engagement. They were given verbal instructions on how to adopt the posture and shown a visual depiction (Figure 1); the words “open” and “closed” were never used, to prevent confounds. The open posture had participants seated with their arms uncrossed and legs uncrossed, while the closed posture had them sit with their arms folded at chest height and legs crossed.

Participants provided all responses verbally, ensuring they maintained their posture throughout the experiment. They read the related instructions and were first presented with an example video. They then viewed the 12 videos (randomized). After each video, participants stated their veracity judgments, replying with a number from a 7-point scale on screen ranging from (−3)—“very dishonest” to 0—“don’t know” to 3—“very honest”. Afterward, they were fully debriefed.

### 3.4. Data Analysis Plan

To investigate the effects of posture on deception detection, two one-way, between-subjects analyses of covariance (ANCOVA) were planned with Posture (Open or Closed) as the between-subjects factor and trait Empathy as the covariate. Two dependent variables (accuracy and bias) are calculated by collapsing participants’ responses on the honesty scale into three possible responses: −3 to −1 were recoded as “lie”, 1 to 3 as “truth”, and 0 as “don’t know”. This is done as differences in response certainty do not reflect differences in accuracy (i.e., “very dishonest” is not more accurate than “dishonest”; for an in-depth discussion on continuous versus dichotomous veracity scales, see [55]). Responses are compared to the veracity of each video and given a score of either correct = 1 or incorrect = 0 and summed across trials; the “don’t know” responses are treated as incorrect.

The coded accuracy data are then processed using Signal Detection Theory (SDT; [56]), to ensure that any effects of the posture manipulation are representative of a change in discriminability and not simply a change in response bias. SDT is recommended as a better method of differentiating the differences in accuracy from response bias when making veracity judgments [5,57]. Two new dependent variables are calculated: A’ measuring overall accuracy independently of bias [58], and B” measuring participants’ response bias [59]. For A’, a value of 0.50 indicates chance level performance. For B”, values < 0 represent a lie bias, while values > 0 indicate a truth bias. Additional one-sample *t*-tests were planned for each posture condition (Open or Closed) to assess judgment bias compared to being unbiased (0).

For the effect of posture on microexpression recognition, the METT scores (%) were to be analyzed using a one-way, between-subjects ANCOVA, with Posture (Open or Closed) as the between-subjects factor and trait Empathy as the covariate. The relationship between deception detection accuracy (overall, lies, and truth) and facial expression recognition are assessed using non-parametric (Spearman’s rho) correlations.

The significance threshold for all analyses was set at *p* < 0.05. The assumptions for the ANCOVAs were verified (i.e., the normality assumption, as assessed by Shapiro–Wilk’s test *p* < 0.05 for all variables, and the homogeneity of variance assumption was met, as assessed by Levene’s test *p* < 0.05 for all comparisons conducted).

All data were analyzed using the open-access statistical software program JASP [60].

## 4. Results

### 4.1. Facial Expression Recognition

An ANCOVA investigated the effect of posture on facial expression recognition, controlling for individual differences in empathy. Contrary to the prediction, the results showed no statistically significant effect of posture, *F* < 1, *p* = 0.987, or empathy, *F*(1, 77) = 1.64, *p* = 0.203, $\eta_{P}^{2}$ = 0.021, on facial recognition performance.

Non-parametric correlations were conducted between facial expression recognition scores and veracity judgments (truths and lies), while considering the posture manipulation. These did not reveal a statistically significant relationship between the two measures either overall (*r*_s_ = 0.13, 95% CI (−0.09, 34), *p* = 0.244, *N* = 80), for Closed posture (*r*_s_ = 16, 95% CI (−0.16, 45), *p* = 324, *N* = 40), or for Open posture (*r*_s_ = 0.15, 95% CI (−0.17, 0.44), *p* = 362, *N* = 40).

### 4.2. Deception Detection

A manipulation check was first conducted to verify that the posture manipulation did not influence uncertainty. An analysis of the “don’t know” responses in each condition was performed, which was not found to be statistically significant, *F* < 1, *p* = 0.812.

The ANCOVA conducted to determine the effect of posture on accuracy (A’), revealed, in line with the prediction, a statistically significant effect of posture (*F*(1, 77) = 5.37, *p* = 0.023, $\eta_{P}^{2}$ = 0.065, 90% CI (0.01, 0.17)), where participants in the Open condition (*M* = 66, *SD* = 19) outperformed those in the Closed condition (*M* = 0.57, SD = 0.21). Empathy was also found to have a statistically significant additive effect (*F*(1, 77) = 5.09, *p* = 0.027, $\eta_{P}^{2}$ = 0.062, 90% CI (0.00, 0.16)).

The ANCOVA conducted to determine if posture affected response bias (B”) did not reveal an effect of posture or empathy, *F*s < 1, *p*s > 0.50. One-sample *t*-tests revealed that judges in both the Open (*M* = 0.62, *SD* = 0.55) and Closed (*M* = 0.54, *SD* = 0.48) postures were significantly truth-biased *t*(39) = 7.15, *p* < 0.001, *d_z_* = 1.13, 95% CI (0.79, 1.46), and *t*(39) = 7.05, *p* < 0.001, *d_z_* = 1.12, 95% CI (0.78, 1.44), respectively.

## 5. Discussion

The posture adopted by judges had a significant effect on their ability to detect deception but not on the accuracy of categorizing microexpressions. Specifically, judges adopting an open posture had higher deception detection scores compared to those in the closed posture. Using a perceptual explanation, the accuracy difference may be a result of posture affecting attention to behavioral information. However, the lack of a difference in facial recognition scores seems to not support this explanation (cf. [34]). Considering a cognitive explanation, posture may be influencing how judges are processing information, such as an increase in resources allocated to the judgment process.

Microexpression recognition, however, was unaffected by either individual differences in trait empathy or posture manipulation. The lack of a posture effect suggests this manipulation does not impact the ability to classify the emotional displays of others. However, microexpression recognition scores in both the open (*M* = 68.58%, *SD* = 16.37%) and closed (*M* = 69.28%, *SD* = 14.66%) postures were relatively high, nearing a ceiling effect, which may have attenuated any embodied effect.

The lack of a relationship between the microexpression scores and empathy may also be explained by the artificial nature of the task (see [61]). Microexpressions are brief flashes of prototypical expressions of emotions, whereas most studies employ recognition tasks using clear valence (positive-negative) facial expressions presented for longer periods (e.g., [45]). Furthermore, recent propositions suggest that empathy relates more to the speed of processing facial expressions, and not how accurately expressions are classified [62]. Importantly, research finds that people are also poor at discriminating genuine from deliberate emotional displays, calling into question their diagnostic value as cues to deception [63,64]. It would be worthwhile to explore if recognition of more naturalistic or social–emotional displays would show an effect of posture.

## 6. Study 2

Study 2 explored the posture effect by considering which aspect of the judges’ judgment was affected by the manipulation. Either open posture judges were more attentive to some behavioral cues (i.e., better detectors) or they were better at processing the information they received (i.e., better analyzers). Judges’ gazing behavior was measured using an eye-tracker to investigate if the posture manipulation changed where they were looking and/or focusing during the veracity judgment process.

We used both high-stakes and low-stakes lie videos in this study for two reasons. Stakes are the rewards to the liar for escaping detection and/or the punishment that they would receive for being caught. Stakes make controlling one’s behavior channels more difficult [35] and increase the likelihood of displaying unintended behavioral cues associated with lying, both in quantity and intensity [36,65], which can make deception detection easier [66,67]. If the posture manipulation affects the attention judges give to behavioral cues a difference in accuracy should be more pronounced for the high-stake lies. The second benefit is uncovering the stability and generalizability of the posture effect on different lie scenarios. It should be noted that the role of stakes in lie detection is a debated topic. The meta-analysis by DePaulo and colleagues [33] reported an effect of motivation (a close proxy to stakes) on the detectability of deception, however, the more recent meta-analysis by Hartwig and Bond [34] failed to replicate this finding.

It was predicted that the posture effect observed in Study 1 would be replicated and extended to the high-stake lies. Second, for the high-stakes condition, this difference between postures would be more pronounced. Finally, it was predicted that posture would influence the gazing behavior of judges.

## 7. Methods

### 7.1. Participants and Design

Thirty-two students (6 males, 26 females; *M_Age_* = 19.88, *SD* = 1.98) were recruited using the university’s online subject pool in return for course credit. A sensitivity analysis for an interaction between Posture (2), Stakes (2), and Veracity (2), given the sample size, estimated that an effect size of Cohen’s *f* = 0.26 (medium-to-large) can be detected with 95% power and an alpha criterion of 0.05. None of the participants had any uncorrected visual impairments.

A mixed-design was employed, with Posture (Open or Closed) as the between-subjects variable, and Veracity (Lies and Truths) and Stakes (High and Low) as the within-subjects variables. The dependent variables were deception detection accuracy, judgment bias, confidence, and eye-gazing behavior. Given Study 1′s accuracy effect and the past literature on the relationship between expansive postures and decision confidence [68,69], the confidence measure was added to assess if the manipulation also influences judges’ self-perceived lie detection ability. Differences in empathy were also measured.

### 7.2. Stimuli and Apparatus

#### 7.2.1. Deception Detection Videos

For the low-stake stimuli, eighteen videos (9 lies and 9 truths) were selected from the BDS (as in Study 1). These are considered to contain low stake lies, as the senders had no incentive to lie and suffered no consequences if caught. For the high-stake stimuli, eighteen videos (9 lies and 9 truths) were selected from Levine’s [70] trivia game interviews. In Levine’s study, students played what they believed was a teamwork game where they could win a cash prize. They worked with a partner, who, unbeknownst to them, was one of the researchers. During the trivia game, the experimenter steps out, leaving the trivia answers unattended. The confederate attempts to convince the participant to cheat. The videos are of the post-trivia game interviews where the participants were asked if they had cheated. Nine videos were of individuals that had cheated, and nine who had not cheated. All videos were on average 20 s long. The videos are considered high stakes as senders believed they were subject to university rules, which if violated could have severe consequences, including expulsion, and if they were successful could win the monetary reward from the game.

#### 7.2.2. Empathy

Trait empathy was assessed using the IRI.

#### 7.2.3. Eye-Tracking

Eye movement was measured using a mobile SensoMotoric Instruments (SMI) RED500 Binocular Eye-Tracker, set to record at 120 Hz. Using a mobile eye-tracker permitted the measuring of eye-gazing behavior without imposing restrictions on movement or causing additional discomfort to participants. The presentation of stimuli, order of the tasks, and recording of eye movements were controlled by the experimenter using the SMI laptop. The videos were displayed to an external monitor (1280 × 960 resolution) with the eye-tracker mounted underneath in a fixed position. For each participant, a 10-point calibration followed by a 4-point validation process was conducted.

### 7.3. Procedure

Participants first completed the IRI. They were then placed in front of the eye-tracker and randomly assigned to a posture condition, open or closed. After ensuring the posture was properly adopted, the calibration and validation of the eye-tracker were performed. Participants were shown instructions regarding the first video set, either high-stakes or low-stakes (counterbalanced, and videos were randomized within each set). For each video, participants gave two verbal responses: the veracity judgment, using the same scale as in Study 1, and their confidence, using a 7-point scale ranging from “not at all confident” to “extremely confident”. At the end, participants were debriefed.

### 7.4. Data Analysis Plan

To investigate the eye-gazing differences between the two postures, the eye-tracking data are coded for several areas of interest (AOI), representing the body (AOI-1), the hands (AOI-2), the upper face (AOI-3), and the lower face (AOI-4). This allows for a fine-grained analysis of the judges’ gazing patterns to different nonverbal channels related to objective and stereotypical cues of deception [32,33]. For each area, two measures from the SMI BeGaze native software are reported: average dwell time as a percentage (AvgDT%), reflecting the time spent looking at the specific area divided by the total looking time of all areas, and fixation count (AvgFC), measuring the average number of times the judges fixated on each target area.

Two mixed multivariate analysis of variance (MANOVA) were planned to analyze the data (for AvgDT% and AvgFC), with Posture (Open or Closed) as the between-subjects factor, Stakes (Low and High) as the within-subjects factor, and the four AOIs (Body, Hands, Face. Upper, Face. Lower) as the dependent measures. Significant effects will be explored with univariate ANOVAs and appropriate post hoc contrasts.

To investigate the effects of posture on deception detection, the judges’ accuracy responses were coded and analyzed using SDT, producing an accuracy (A’) and bias (B”) variable. Two two-way mixed ANCOVAs are planned with Posture (Open or Closed) as the between-subjects factor, and Stakes (High and Low) as the within-subjects factor, while controlling for individual differences in empathy. Significant effects will be analyzed using post hoc contrasts.

To explore the differences in judgment confidence based on posture, between-subjects *t*-tests are planned, alongside non-parametric correlations exploring the relationship between confidence ratings and accuracy scores (overall and based on veracity).

Data were processed and analyzed using the SMI BeGaze software tool and the statistical program JASP.

## 8. Results

### 8.1. Eye-Movement

A MANOVA was considered for analyzing the eye gazing data, however, a preliminary inspection revealed that several dependent measures were either correlated too strongly (*r*s > 0.9; multicollinearity) or not at all (*r*s < 0.2), and several assumptions for this analysis were violated (i.e., homogeneity of covariance, Box’s M, *p*s < 0.001; multivariate normality, Royston’s Test, *p*s < 0.001). An inspection of the skewness and kurtosis, in addition to Q–Q plots and histograms, indicated that the assumption of normality of variance was violated for several AOIs. Therefore, the appropriate dependent variables were transformed to obtain a normal distribution of data and analyzed using between-subjects *t*-tests.

**Figure 2:**
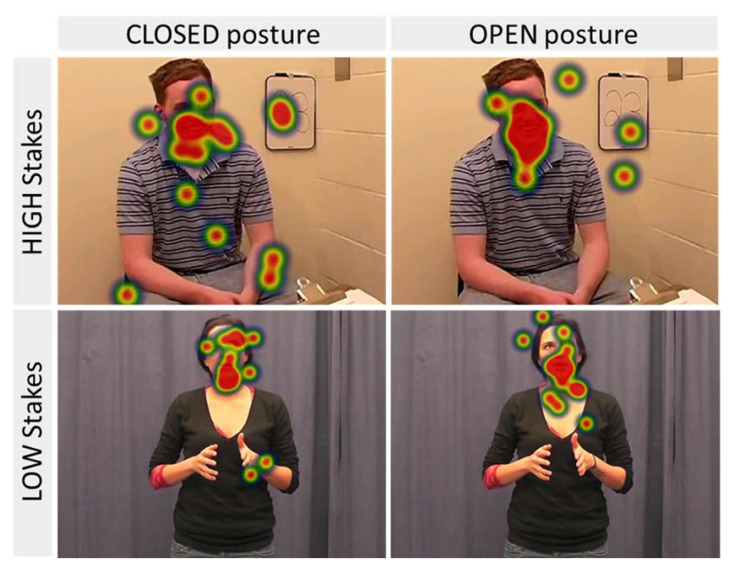
Eye-gaze heat maps for looking frequency of Closed and Open posture judges looking at the High-Stakes and Low-Stakes videos. All subjects agreed to the publication of their images.

Preliminary analysis revealed differences between the high-stakes and low-stakes videos for both variables (stemming from the differences between the videos sets; see Figure 2), *p*s < 0.001, however, no interactions with the other factors were found, and analyzing them separately did not impact the results; therefore, Stakes was collapsed as a factor. Empathy, also, did not show any effects on looking patterns, *F*s < 1, *p*s > 0.14.

An independent samples t-test comparing the total (combined AOIs) percentage of looking times (AvgDT%) based on posture was conducted. Overall, the judges in the Open posture (*M* = 18.6%, *SD* = 4.7%) devoted less time looking at senders, than those in the Closed posture (*M* = 21.8%, *SD* = 1.5%). To obtain normality, the analysis was conducted on base 10 log-transformed data. This revealed a statistically significant difference based on posture, *t*(24.67) = 2.08, *p* = 0.048, *d_s_* = 0.72, 95% CI (0.01, 1.44) (unequal variance). 

To unpack this difference, planned comparisons were conducted for each AOI. Investigating the differences in looking at the hands of senders revealed that the Open posture judges (*M* = 1.8%, *SD* = 1.1%) looked less at this area than the Closed posture judges (*M* = 3.2%, *SD* = 1.8%), *t*(30) = −2.40, *p* = 0.023, *d_s_* = −0.85, 95% CI (−1.57, −0.12) (square root-transformed data). No other differences in the judges’ gazing behavior between the two posture conditions were found, *t*s ≤ 1, *p*s > 0.05.

**Figure 3:**
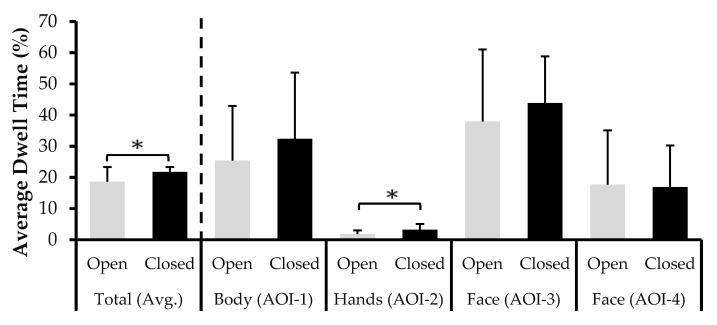
Average dwell time as a percentage of total looking time for each AOI split by judge Posture (error bars ± 1 SD). The line over the bars represents a significant difference (* *p* < 0.05).

To understand if judges differed in the number of times they focused on different areas, and not just the amount of time spent, analyses were conducted on the fixation count of each AOI. Overall, the judges in both conditions seemed to fixate an equal number of times on the senders while making their veracity judgments, *t*(30) = −1.01, *p* = 0.321. Considering each AOI separately, the planned *t*-tests revealed a marginally significant effect for the Open posture judges (*M* = 1.3, *SD* = 0.9) fixating fewer times at the hands than the Closed posture judges (*M* = 2.2, *SD* = 1.6), *t*(30) = −1.84, *p* = 0.076, *d_s_* = −0.65, 95% CI (−1.36, 0.07) (square root-transformed data). No other significant differences emerged (*t*s ≤ 1, *p*s > 0.05). See Figure 3.

### 8.2. Deception Detection

A repeated-measures ANCOVA was conducted on Posture (Open or Closed) and Stakes (High and Low) while controlling for individual differences in empathy. A marginal main effect of stakes was observed (*F*(1, 29) = 4.04, *p* = 0.053, $\eta_{P}^{2}\;$= 0.119) suggesting that the High-Stakes videos (*M* = 0.61, *SD* = 0.16) were easier to classify than the Low-stakes videos (*M* = 0.51, *SD* = 0.21). While judges in the Open posture (*M* = 0.59, *SD* = 0.15) had higher accuracy than judges in the Closed posture (*M* = 0.55, *SD* = 0.11), the difference was not statistically significant (*F* < 1, *p* = 0.335). The Stakes X Posture interaction, while also in the predicted direction—the difference between the Open and Closed posture on accuracy being more pronounced for the High-Stakes videos (*M_diff_* = 5.15%) than the Low-Stakes videos (*M_diff_* = 3.60%)—was not statistically significant (*F* < 1, *p* = 0.948). Empathy was not found to affect the results (*F*s < 1, *p* = 0.644).

Subsequently, an ANCOVA investigated the influence of posture and stakes on response bias (B”), controlling for empathy. This did not indicate any effect of posture (*F* < 1, *p* = 0.693, stakes, *F* < 1, *p* = 0.956) or interaction (*F*(1, 28) = 2.60, *p* = 0.118, $\eta_{P}^{2}$ = 0.085). All judges were significantly truth-biased, both in the Open (*M* = 0.44, *SD* = 0.35; *t*(16) = 5.24, *p* < 0.001, *d_z_* = 1.27, 95% CI (0.71, 1.80)), and Closed postures (*M* = 0.36, *SD* = 0.25; *t*(14) = 5.56, *p* < 0.001, *d_z_* = 1.44, 95% CI (0.81, 2.03)).

#### Mini-Meta Analysis

Given the lower statistical power to detect the posture-accuracy effect of interest in Study 2, we meta-analyzed the effect of Posture (Open or Closed) of our two studies using a sample-size weighted fixed-effects analysis with unbiased mean effect size estimates (i.e., Hedge’s *g*; Study 1, *g* = 0.45, 95% CI (0.01, 0.89), Study 2, *g* = 0.29, 95% CI (−0.40, 0.99)) [71]. The effect was statistically significant (M*_g_* = 0.40, 95% CI (0.03, 0.78), Z = 2.10, *p* = 0.036 (two-tailed)), such that the judges in the Open posture were more accurate at detecting deception than the judges in the Closed posture.

### 8.3. Confidence

An independent samples *t*-test was conducted on the judges’ confidence ratings, based on posture. The analyses did not find a statistically significant difference (*t*(30) = 1.23, *p* = 0.227), suggesting that the posture manipulation may not impact judges’ confidence in their veracity judgments.

The non-parametric correlations for confidence ratings and veracity judgment did not reveal any relationships either with the overall accuracy (*r*_s_ = −0.14, 95% CI (−0.47, 0.22), *p* = 0.433, *N* = 32), the truth accuracy (*r*_s_ = 0.10, 95% CI (−0.25, 0.44), *p* = 0.570, *N* = 32), or the lie accuracy (*r*_s_ = 0.10, 95% CI (−0.26, 0.43), *p* = 0.607, *N* = 32).

## 9. Discussion

The analysis comparing looking patterns revealed an additional effect of the posture manipulation on judges, which may assist in understanding the elements influencing veracity judgments seen in Study 1. The judges who adopted an open posture looked less at senders overall (both in the number of times and in duration) than the judges adopting a closed posture. This seems to contradict the social acuity assumption that adopting an open posture will make judges more attentive and/or receptive to the behaviors of others. Specifically, the data show that judges in the open posture looked less at the hands of senders than the judges in the closed posture, but focused equally on all other locations (i.e., face, arms, body). This finding seems to also contradict the emotion-based lie detection literature, which claims that astute judges focus more on the facial expressions (e.g., microexpressions) of senders. Here, the opposite is displayed, as the judges in the open posture focused less overall on the nonverbal behavior of senders.

The posture–accuracy effect did not replicate in Study 2 (attributed to the smaller sample size and more uncertainty in parameter estimation, i.e., wider confidence interval), although, a tentative trend in the same direction was observed in the data which was bolstered by the mini-meta-analysis combining the effects sizes showing support for the existence of a moderately sized effect. The data also suggested a slight difference in performance due to the type of lie judges saw, as evidenced by the marginal stakes effect on accuracy. This would indicate that the High-Stakes videos were easier to classify than the Low-Stakes videos. However, the data do not permit any strong inferences with the current sample.

Finally, the results showed that posture did not affect judges’ response bias (replicating Study 1) or confidence in their veracity judgments. These findings are noteworthy, as past intervention studies (e.g., lie detection training) have been found to occasionally result in detrimental effects on the judges’ biases [5] and confidence [3]. This suggests that the posture manipulation may be useful as a passive lie detection technique to improve deception detection accuracy without the need for training or lengthy and complex a priori preparation of the environment.

## 10. General Discussion

Over two experiments, we investigated a novel effect of adopting different postures on judges’ perceptions and judgments of liars and truth-tellers. The data find that the posture adopted by a judge impacted their ability to discriminate truthful and deceptive statements (Study 1) and their gazing behavior (Study 2) without affecting response bias or confidence.

In Study 1, it was found that adopting an open posture resulted in a higher discriminability compared to adopting a closed posture, an effect that was more visible with higher levels of empathy. The results also showed that this effect was not attributable to a shift in response bias brought about by the manipulation. Facial expression recognition was unaffected by either empathy or the posture manipulation.

Building on these results, in Study 2, the looking behavior of the judges in the two posture conditions was assessed using a mobile eye-tracker while also expanding the deception detection task to high-stakes stimuli, assessing the stability and generalizability of the posture effect on different types of lies. With respect to the judgment data, judges adopting an open posture showed a trend towards higher accuracy compared to their closed posture counterparts. For stakes, a marginal improvement based on posture was found, where higher-stakes lies and truths were easier to classify than their lower-stakes counterparts. Adopting an open posture also increased the difference in detection scores between the two lie types, although this trend was not statistically significant.

Concerning gazing behavior, adopting an open posture resulted in less attention being given to the nonverbal behavior of senders, contrasting what the literature would predict. It was found that judges placed in an open posture spent less time gazing at senders. Specifically, they focused less on the hands of senders. Potentially, open posture judges were faster and/or more efficient at extracting information from nonverbal signals and thus required less time looking at senders, or they relied less on nonverbal information for their veracity judgments.

The case for an information-processing account can be made by combining the results of the two studies. In Study 1, the accuracy and empathy effects can be taken as posture affecting the processing of information more than the attention paid to the senders’ cues. Indeed, the compatibility between posture and situation has been shown to facilitate performance by increasing the availability of processing resources [22,72]. In Study 2, the reduced gazing time for open posture judges would suggest that information processing, and not attention, is impacted by the posture effect. Perhaps, if people adopt an open posture, they are primed to dedicate more resources to processing social information. In this way, posture may be affecting how the judges process information from senders, and not their tendency to inspect their targets more thoroughly. Indeed, past research has shown that relying more on other channels (e.g., paraverbal or content) can increase accuracy [73]. Reciprocally, the difference in dwell time can be interpreted as the closed posture judges being less actively engaged in the task, adopting a more relaxed and passive attitude to the decoding task (i.e., lingering). Unfortunately, the settings used for the eye tracker (specifically, the low sample rate) do not permit such an exploration of the data, but this merits future consideration.

Alternatively, in keeping with the “openness to communication” dimension, closed posture judges may be focusing more on senders’ hands in an attempt to avoid areas associated with social information and interaction [74]. For instance, there are cultural differences in the communicative value of hand gestures and their support for verbal communication [75]. However, this does not account for the lack of differences in looking at the face between the two posture conditions. An alternative explanation may be that gesticulation was deemed more important in the closed posture condition, placing greater emphasis on message understanding than on other nonverbal information [76]. Extensions of the current work should explore the different communicative channels that the judges considered and determine their individual effects on veracity judgments.

The present two-posture paradigm limits any directionality interpretation relating to the increasing or decreasing accuracy compared to a non-intervention judgment scenario. However, tentatively, the accuracy seen in the open posture (66% in Study 1; 59% in Study 2) would suggest that the manipulation aided deception detection, as the closed posture accuracy (57% in Study 1; 55% in Study 2) is in line with the levels usually reported in past research (i.e., 54%; [4,33]). This suggests that the current passive lie detection approach produces a moderate effect-sized improvement on accuracy, similar to that obtained from active deception detection training approaches [77]. This effect, if replicated, has practical implications for security, law enforcement, and forensic settings. For example, in many countries, a police suspect cannot be forcefully interrogated using a lie detection technique (such as the cognitive lie detection method; [6]). However, using the current paradigm, interviewers can simply adopt an open posture to improve their odds of discriminating veracity, without becoming more biased or overconfident.

The postural effect also has implications for our understanding of nonverbal behavior in social interactions and embodied cognition. Due to the commonality of such postures in daily life, their effects on perception are important to understand. Individuals adopt these postures frequently, spontaneously, and unconsciously, which may unknowingly affect their interactions with and perceptions of others in social settings. The current findings provide the initial evidence for a new embodied effect of postures on judgment under uncertainty (here, deception detection).

### 10.1. Limitations

Considering the exploratory nature of the current research, a few limitations must be highlighted. First, contrasting the assumed effect sizes of interest for the posture manipulation, the results indicate that although a moderate effect of posture may exist (as suggested by the mini-meta-analysis) it is also highly variable (i.e., the margin of error is 0.38). This variability may explain the difference between the two studies and suggest that replication attempts must consider a smaller real-world effect [78], requiring a larger sample size (and statistical power) to adequately investigate.

Second, a limitation of the current methodology is the inability to measure emotion-specific recognition rates, hindering our ability to explore the relationship between veracity judgment accuracy and microexpression recognition. Speculatively, although a difference was not observed overall, different emotions (and valences) have been shown to produce different recognition rates [79], thus, using aggregate recognition scores may obscure the relevant effects (e.g., the open posture improving the recognition of positive emotions that relate to communication, but hindering the recognition of negative emotions). Replications should consider employing a more detailed approach considering individual recognition rates and processing speed on a per emotion basis.

Third, the study did not consider a priori specific sender–judge effects on judgments, such as the gender of the expressors (i.e., in the facial recognition task) or senders (i.e., in the deception detection task) or the demographics of the judges. While such factors have not been found to impact the accuracy of veracity judgments [1], for facial expression recognition it may be a pertinent factor to consider [80]. Such nuanced explorations merit consideration given the importance of demeanor in veracity judgments [42].

Fourth, the experiences of judges in the two postures were not measured. This limits our ability to interpret the conscious effects that adopting these postures may have had on judges (e.g., discomfort) and on their decision-making process. Replications should incorporate more self-report and physiological measures from judges.

### 10.2. Future Directions

The present approach focused on factors that have strong theoretical links to the deception literature (e.g., empathy and microexpression recognition; [42]). On the basis of our findings, future explorations should consider additional individual differences which may expand on and clarify the current effects, giving more emphasis to potential demographic factors. Gender differences may be relevant in exploring the gazing and nonverbal sensitivity relationships in more detail [80], as would expanding to other traits related to nonverbal sensitivity, such as emotional intelligence. Similarly, the emphasis on sender message content should be considered more strongly, as the current findings suggest that a substantial weight was placed on the senders’ statements as opposed to their nonverbal behavior; indeed, message content has been argued by some scholars as a better source of diagnostic veracity information [76].

An important extension of the current paradigm would be to incorporate more interactive designs, as many veracity judgments in forensic settings are done on the basis of face-to-face interactions [81]. In an optimistic prediction, the open posture effect may foster an affiliation between the sender and the judge [82], leading to similar embodied states (i.e., exchanging information accurately, especially for truth-tellers), activating similar cognitive and affective processes [83], enhancing empathy and rapport [84], and improving communication. Of note, the posture effects can be bidirectional, influencing both the adopter and the observer, which may produce additional effects of interest, such as increased impression management by liars or less critical appraisals by judges, as proposed by the Interpersonal Deception Theory (IDT) framework [85].

As a general recommendation, future explorations of the posture effect should employ a larger sample size and include scenarios containing more serious (e.g., criminal) lies to fully understand the role that the judge’s posture can have on veracity judgments and the generalizability of the posture effects. Considerations may also be given to expanding the postures paradigm to non-deception and more naturalistic scenarios.

## 11. Conclusions

At present, this is the first exploration into the effect of posture on deception detection accuracy, extending the work on embodiment to include veracity judgment and eye-gazing differences. The results offer preliminary support for a novel postural effect on deception detection. The data show that the posture adopted by a judge can produce significant differences in gazing behavior and deception detection. The effect also indicates a relationship with empathic perception and information processing, suggesting that the posture manipulation relates to how information is processed and not to the attention given to specific behavior. The aim of this approach was to aid deception detection without introducing harmful elements or additional demand characteristics into the decoding process. From an applied setting, the posture manipulation may offer a simple-to-implement method for improving the accuracy of veracity judgments.

## Data Availability

The data presented in this study are available on request from the corresponding author.

## References

[1] Aamodt M.G., Custer H. (2006). Who Can Best Catch a Liar?. Forensic Exam..

[2] Bond C.F., DePaulo B.M. (2006). Accuracy of Deception Judgments. Personal. Soc. Psychol. Rev..

[3] Holm H.J., Kawagoe T. (2010). Face-to-Face Lying—An Experimental Study in Sweden and Japan. J. Econ. Psychol..

[4] Levine T.R. (2020). Duped: Truth-Default Theory and the Social Science of Lying and Deception.

[5] Meissner C.A., Kassin S.M. (2002). “He’s Guilty!”: Investigator Bias in Judgments of Truth and Deception. Law Hum. Behav..

[6] Vrij A. (2008). Detecting Lies and Deceit: Pitfalls and Opportunities.

[7] Funk F., Todorov A. (2013). Criminal Stereotypes in the Courtroom: Facial Tattoos Affect Guilt and Punishment Differently. Psychol. Public Policy Law.

[8] Levine T.R., Serota K.B., Shulman H., Clare D.D., Park H.S., Shaw A.S., Shim J.C., Lee J.H. (2011). Sender Demeanor: Individual Differences in Sender Believability Have a Powerful Impact on Deception Detection Judgments. Hum. Commun. Res..

[9] Beigel H.G. (1952). The Influence of Body Position on Mental Processes. J. Clin. Psychol..

[10] Neumann R., Förster J., Strack F., Musch J., Klauer K.C. (2003). Motor compatibility: The bidirectional link between behavior and evaluation. The Psychology of Evaluation: Affective Processes in Cognition and Emotion.

[11] Ekman P., Friesen W.V. (1967). Head and Body Cues in the Judgment of Emotion: A Reformulation. Percept. Mot. Ski..

[12] Mehrabian A., Friar J.T. (1969). Encoding of Attitude by a Seated Communicator via Posture and Position Cues. J. Consult. Clin. Psychol..

[13] D’Mello S., Chipman P., Graesser A. (2007). Posture as a Predictor of Learner’s Affective Engagement. Proc. Ann. Meet. Cogn. Sci. Soc..

[14] Price T.F., Harmon-Jones E. (2010). The Effect of Embodied Emotive States on Cognitive Categorization. Emotion.

[15] Riskind J.H., Gotay C.C. (1982). Physical Posture: Could It Have Regulatory or Feedback Effects on Motivation and Emotion?. Motiv. Emot..

[16] Stepper S., Strack F. (1993). Proprioceptive Determinants of Emotional and Nonemotional Feelings. J. Personal. Soc. Psychol..

[17] Carney D.R., Cuddy A.J.C., Yap A.J. (2015). Review and Summary of Research on the Embodied Effects of Expansive (vs. Contractive) Nonverbal Displays. Psychol. Sci..

[18] Ranehill E., Dreber A., Johannesson M., Leiberg S., Sul S., Weber R.A. (2015). Assessing the Robustness of Power Posing. Psychol. Sci..

[19] Simmons J.P., Simonsohn U. (2017). Power Posing: P-Curving the Evidence. Psychol. Sci..

[20] Nair S., Sagar M., Sollers J., Consedine N., Broadbent E. (2015). Do Slumped and Upright Postures Affect Stress Responses? A Randomized Trial. Health Psychol..

[21] Wilkes C., Kydd R., Sagar M., Broadbent E. (2017). Upright Posture Improves Affect and Fatigue in People with Depressive Symptoms. J. Behav. Ther. Exp. Psychiatry.

[22] Riskind J.H. (1983). Nonverbal Expressions and the Accessibility of Life Experience Memories: A Congruence Hypothesis. Soc. Cogn..

[23] Michalak J., Mischnat J., Teismann T. (2014). Sitting Posture Makes a Difference-Embodiment Effects on Depressive Memory Bias. Clin. Psychol. Psychother..

[24] Veenstra L., Schneider I.K., Koole S.L. (2017). Embodied Mood Regulation: The Impact of Body Posture on Mood Recovery, Negative Thoughts, and Mood-Congruent Recall. Cogn. Emot..

[25] Wood J.V., Saltzberg J.A., Goldsamt L.A. (1990). Does Affect Induce Self-Focused Attention?. J. Personal. Soc. Psychol..

[26] Keltner D., Gruenfeld D.H., Anderson C. (2003). Power, Approach, and Inhibition. Psychol. Rev..

[27] Anderson C., Galinsky A.D. (2006). Power, Optimism, and Risk—taking. Eur. J. Soc. Psychol..

[28] Anderson C., Berdahl J.L. (2002). The Experience of Power: Examining the Effects of Power on Approach and Inhibition Tendencies. J. Personal. Soc. Psychol..

[29] Schwarz N., Clore G.L., Higgins E.T., Kruglanski A. (1996). Feelings and phenomenal experiences. Social Psychology: Handbook of Basic Principles.

[30] Machotka P. (1965). Body Movement as Communication. Dialogues Behav. Sci. Res..

[31] Bogaard G., Meijer E.H., Vrij A., Merckelbach H. (2016). Strong, but Wrong: Lay People’s and Police Officers’ Beliefs about Verbal and Nonverbal Cues to Deception. PLoS ONE.

[32] (2006). The Global Deception Research Team A World of Lies. J. Cross-Cult. Psychol..

[33] DePaulo B.M., Lindsay J.J., Malone B.E., Muhlenbruck L., Charlton K., Cooper H. (2003). Cues to Deception. Psychol. Bull..

[34] Hartwig M., Bond C.F. (2014). Lie Detection from Multiple Cues: A Meta-Analysis. Appl. Cogn. Psychol..

[35] Ekman P. (2003). Darwin, Deception, and Facial Expression. Ann. N. Y. Acad. Sci..

[36] Zloteanu M., Freitas-Magalhaes A., Borod J. (2020). Reconsidering facial expressions and deception detection. Handbook of Facial Expression of Emotion.

[37] Frank M.G., Svetieva E., Mandal M.K., Awasthi A. (2015). Microexpressions and deception. Understanding Facial Expressions in Communication: Cross-Cultural and Multidisciplinary Perspectives.

[38] Zloteanu M., Williams E., Sheeha I. (2015). The role of emotions in detecting deception. Deception: An Interdisciplinary Exploration.

[39] Matsumoto D., Hwang H.C., Skinner L.G., Frank M.G. (2014). Positive Effects in Detecting Lies from Training to Recognize Behavioral Anomalies. J. Police Crim. Psychol..

[40] Warren G., Schertler E., Bull P. (2009). Detecting Deception from Emotional and Unemotional Cues. J. Nonverbal Behav..

[41] Jordan S., Brimbal L., Wallace D.B., Kassin S.M., Hartwig M., Street C.N.H. (2019). A Test of the Micro-Expressions Training Tool: Does It Improve Lie Detection?. J. Investig. Psychol. Offender Profiling.

[42] Zloteanu M., Bull P., Krumhuber E.G., Richardson D.C. (2021). Veracity Judgement, Not Accuracy: Reconsidering the Role of Facial Expressions, Empathy, and Emotion Recognition Training on Deception Detection. Q. J. Exp. Psychol..

[43] Alpers G.W., Gerdes A. (2007). Here Is Looking at You: Emotional Faces Predominate in Binocular Rivalry. Emotion.

[44] Vuilleumier P., Armony J.L., Driver J., Dolan R.J. (2001). Effects of Attention and Emotion on Face Processing in the Human Brain: An Event-Related FMRI Study. Neuron.

[45] Carr M.B., Lutjemeier J.A. (2005). The Relation of Facial Affect Recognition and Empathy to Delinquency in Youth Offenders. Adolescence.

[46] Gery I., Miljkovitch R., Berthoz S., Soussignan R. (2009). Empathy and Recognition of Facial Expressions of Emotion in Sex Offenders, Non-Sex Offenders and Normal Controls. Psychiatry Res..

[47] Dimberg U., Andréasson P., Thunberg M. (2011). Emotional Empathy and Facial Reactions to Facial Expressions. J. Psychophysiol..

[48] Faul F., Erdfelder E., Lang A.-G., Buchner A. (2007). G* Power 3: A Flexible Statistical Power Analysis Program for the Social, Behavioral, and Biomedical Sciences. Behav. Res. Methods.

[49] Street C.N., Tbaily L., Baron S., Khalil-Marzouk P., Hanby B., Wright K., Richardson D.C. Bloomsbury Deception Set. Proceedings of the British Psychological Society Division of Forensic Psychology Conference.

[50] Ekman P. (2002). Microexpression Training Tool, Subtle Expression Training Tool.

[51] Matsumoto D., LeRoux J., Wilson-Cohn C., Raroque J., Kooken K., Ekman P., Yrizarry N., Loewinger S., Uchida H., Yee A. (2000). A New Test to Measure Emotion Recognition Ability: Matsumoto and Ekman’s Japanese and Caucasian Brief Affect Recognition Test (JACBART). J. Nonverbal. Behav..

[52] Davis M.H. (1983). Measuring Individual Differences in Empathy: Evidence for a Multidimensional Approach. J. Personal. Soc. Psychol..

[53] Davis M.H., Franzoi S.L. (1991). Stability and Change in Adolescent Self-Consciousness and Empathy. J. Res. Personal..

[54] Bartholow B.D., Sestir M.A., Davis E.B. (2005). Correlates and Consequences of Exposure to Video Game Violence: Hostile Personality, Empathy, and Aggressive Behavior. Personal. Soc. Psychol. Bull..

[55] Levine T.R. (2001). Dichotomous and Continuous Views of Deception: A Reexamination of Deception Ratings in Information Manipulation Theory. Commun. Res. Rep..

[56] Green D., Swets J. (1966). Signal Detection Theory and Psychophysics.

[57] Masip J., Alonso H., Garrido E., Herrero C. (2009). Training to Detect What? The Biasing Effects of Training on Veracity Judgments. Appl. Cogn. Psychol..

[58] Rae G. (1976). Table of A’. Percept. Mot. Ski..

[59] Donaldson W. (1992). Measuring Recognition Memory. J. Exp. Psychol. Gen..

[60] JASP Team (2020). JASP (Version 0.14.1)[Computer Software]. https://jasp-stats.org/faq/how-do-i-cite-jasp/.

[61] Zloteanu M., Krumhuber E.G. (2021). Expression Authenticity: The Role of Genuine and Deliberate Displays in Emotion Perception. Front. Psychol..

[62] Kosonogov V., Titova A., Vorobyeva E. (2015). Empathy, but Not Mimicry Restriction, Influences the Recognition of Change in Emotional Facial Expressions. Q. J. Exp. Psychol..

[63] Zloteanu M., Krumhuber E.G., Richardson D.C. (2018). Detecting Genuine and Deliberate Displays of Surprise in Static and Dynamic Faces. Front. Psychol..

[64] Zloteanu M., Krumhuber E.G., Richardson D.C. (2021). Acting Surprised: Comparing Perceptions of Different Dynamic Deliberate Expressions. J. Nonverbal. Behav..

[65] Hartwig M., Bond C.F. (2011). Why Do Lie-Catchers Fail? A Lens Model Meta-Analysis of Human Lie Judgments. Psychol. Bull..

[66] Frank M.G., Feeley T.H. (2003). To Catch a Liar: Challenges for Research in Lie Detection Training. J. Appl. Commun. Res..

[67] Porter S., ten Brinke L.M., Wallace B. (2012). Secrets and Lies: Involuntary Leakage in Deceptive Facial Expressions as a Function of Emotional Intensity. J. Nonverbal. Behav..

[68] Briñol P., Petty R.E., Valle C., Rucker D.D., Becerra A. (2007). The Effects of Message Recipients’ Power before and after Persuasion: A Self-Validation Analysis. J. Personal. Soc. Psychol..

[69] Fischer J., Fischer P., Englich B., Aydin N., Frey D. (2011). Empower My Decisions: The Effects of Power Gestures on Confirmatory Information Processing. J. Exp. Soc. Psychol..

[70] Levine T.R. (2007). MSU Trivia Game Interviews.

[71] Goh J.X., Hall J.A., Rosenthal R. (2016). Mini Meta-Analysis of Your Own Studies: Some Arguments on Why and a Primer on How: Mini Meta-Analysis. Soc. Personal. Psychol. Compass.

[72] Förster J., Strack F. (1996). Influence of Overt Head Movements on Memory for Valenced Words: A Case of Conceptual-Motor Compatibility. J. Personal. Soc. Psychol..

[73] Bond C.F., Howard A.R., Hutchison J.L., Masip J. (2013). Overlooking the Obvious: Incentives to Lie. Basic Appl. Soc. Psychol..

[74] Mansell W., Clark D.M., Ehlers A., Chen Y.-P. (1999). Social Anxiety and Attention Away from Emotional Faces. Cogn. Emot..

[75] Beattie G., Shovelton H. (1999). Mapping the Range of Information Contained in the Iconic Hand Gestures That Accompany Spontaneous Speech. J. Lang. Soc. Psychol..

[76] Levine T.R. (2015). New and Improved Accuracy Findings in Deception Detection Research. Curr. Opin. Psychol..

[77] Driskell J.E. (2012). Effectiveness of Deception Detection Training: A Meta-Analysis. Psychol. Crime Law.

[78] Simonsohn U. (2015). Small Telescopes: Detectability and the Evaluation of Replication Results. Psychol. Sci..

[79] Nelson N.L., Russell J.A. (2013). Universality Revisited. Emot. Rev..

[80] Hall J.A., Gunnery S.D., Horgan T.G., Hall J.A., Schmid Mast M., West T.V. (2016). Gender differences in interpersonal accuracy. The Social Psychology of Perceiving Others Accurately.

[81] Gudjonsson G.H. (1992). The Psychology of Interrogations, Confessions and Testimony.

[82] Lakin J.L., Jefferis V.E., Cheng C.M., Chartrand T.L. (2003). The Chameleon Effect as Social Glue: Evidence for the Evolutionary Significance of Nonconscious Mimicry. J. Nonverbal. Behav..

[83] Barsalou L.W., Niedenthal P.M., Barbey A.K., Ruppert J.A. (2003). Social Embodiment. Psychol. Learn. Motiv..

[84] Stel M., Van Baaren R.B., Vonk R. (2008). Effects of Mimicking: Acting Prosocially by Being Emotionally Moved. Eur. J. Soc. Psychol..

[85] Burgoon J.K., Levine T.R., Smith S.W., Wilson S.R. (2010). Advances in Deception Detection. New Directions in Interpersonal Communication Research.

